# Comprehensive Literature Review on Melanoma of Unknown Primary Site Triggered by an Intriguing Case Report

**DOI:** 10.3390/diagnostics14192210

**Published:** 2024-10-03

**Authors:** Eliza-Maria Bordeanu-Diaconescu, Andrei Cretu, Andreea Grosu-Bularda, Mihaela-Cristina Andrei, Florin-Vlad Hodea, Catalina-Stefania Dumitru, Valentin Enache, Cosmin-Antoniu Creanga, Ioan Lascar, Cristian-Sorin Hariga

**Affiliations:** 1 Department of Plastic Surgery and Reconstructive Microsurgery, “Carol Davila” University of Medicine and Pharmacy Bucharest, 010825 Bucharest, Romania; eliza.diaconescu@umfcd.ro (E.-M.B.-D.); andreea.grosu-bularda@umfcd.ro (A.G.-B.); cristian.hariga@umfcd.ro (C.-S.H.); 2 Burn Centre, Emergency Clinical Hospital of Bucharest, 014461 Bucharest, Romania; 3 Department of Anatomical Pathology, Clinical Emergency Hospital of Bucharest, 014461 Bucharest, Romania

**Keywords:** MUP, melanoma of unknown primary, non-cutaneous melanoma, metastatic melanoma

## Abstract

Melanoma is one of the most aggressive forms of skin cancer. While most melanomas have a discernible primary site, a small subset, approximately 3.2%, present as a metastatic disease without an identifiable primary origin, a condition known as melanoma of unknown primary (MUP). Unusual cases of primary melanoma have also been previously reported in the respiratory, gastrointestinal, and urogenital tracts. MUP typically is found in lymph nodes, subcutaneous sites, and visceral organs, with hypotheses about its origin including spontaneous primary tumor regression and ectopic melanocytes. MUP presents unique challenges in diagnosis and treatment due to the absence of a detectable primary tumor. Understanding its genetic and molecular features, epidemiology, prognostic factors, and treatment options is crucial for optimizing patient care and outcomes in this subset of melanoma patients. We conducted an extensive literature review triggered by a case report of a patient with suspected MUP. A 51-year-old woman was transferred from another hospital where an incision was performed for a suspected superinfected hematoma of the left thigh. Since the patient showed high leukocytosis and redness and swelling of the thigh, local debridement, drainage, and excisional biopsy of the tumor mass were performed in our unit in the emergency setting, and the tumor was taken for histopathology evaluation. Intraoperatively, the mass appeared nonspecific. The permanent histopathology report established a diagnosis of melanoma, with tumor proliferation also involving lymphoid tissue, and despite broad clinical and imagistic assessments, the primary melanoma could not be identified. Clinicians must be aware of the varied clinical manifestations of malignant melanoma, especially in cases of occult melanoma where the primary site is not evident.

## 1. Introduction

Melanoma, a malignant tumor of melanocytes, is one of the most aggressive forms of skin cancer. While the majority of melanomas have a discernible primary site, a small subset, approximately 3.2%, present as a metastatic disease without an identifiable primary origin, a condition known as melanoma of unknown primary (MUP). Melanoma of unknown primary is an uncommon type of melanoma, predominantly diagnosed in the subcutaneous tissue and lymph nodes, but it can also be found in other soft tissues or even in various visceral sites. MUP often poses significant diagnostic and therapeutic challenges due to its atypical presentation [1,2].

Das Gupta formally described the entity of MUP in 1963. His proposed diagnostic criteria excluded patients who had any of the following: a history of orbital exenteration or enucleation; a history of excision or the removal of any mole, birthmarks, freckles, chronic paronychia, or skin blemishes; the presence of a scar in the skin drained by the nodal basin containing metastatic melanoma; or an incomplete physical examination (including ophthalmoscopy and the examination of the anus and genitalia) [3].

The etiology of MUP remains elusive, with several hypotheses proposed to explain its origin. These include the possibility of the spontaneous regression of the primary tumor, leaving behind metastatic deposits, and the presence of ectopic melanocytes within lymph nodes or visceral organs that give rise to melanoma. Understanding the behavior and origin of MUP is critical for developing targeted treatment strategies and improving patient outcomes [2,3].

It is crucial to thoroughly investigate all the potential sites before diagnosing MUP, as some hidden locations, such as subungual melanoma, may be overlooked. For instance, Rousset et al. reported a case initially diagnosed as lymph node MUP, which was later identified as a regressed amelanotic melanoma of the nail, indicating that MUP was incorrectly diagnosed [4]. However, it is important to keep in mind that, even when finding a lesion resembling melanoma in a clinical examination, it still might not be the primary tumor, but only a cutaneous metastasis of another cutaneous melanoma. Out of all the cutaneous metastases arising from different malignancies, around 75% are melanoma metastases, arising either near the primary tumor (locoregional metastasis) or at a more distant site, so it is important for the dermatologist to differentiate between the different lesions in a dermoscopy [5].

In this article, we present a detailed case report of a patient with suspected MUP, followed by a comprehensive review of the literature. We aim to shed light on the diagnostic complexities associated with managing this rare and complex condition. Through this exploration, we hope to contribute to the existing knowledge base and offer insights that may guide future research and clinical practice in the management of MUP.

## 2. Case Presentation

A 51-year-old woman was presented to our emergency department after being discharged upon request from another hospital, where surgical intervention was performed 2 days prior due to a suspected superinfected hematoma of the left thigh, and antibiotic therapy was initiated.

**Figure 1:**
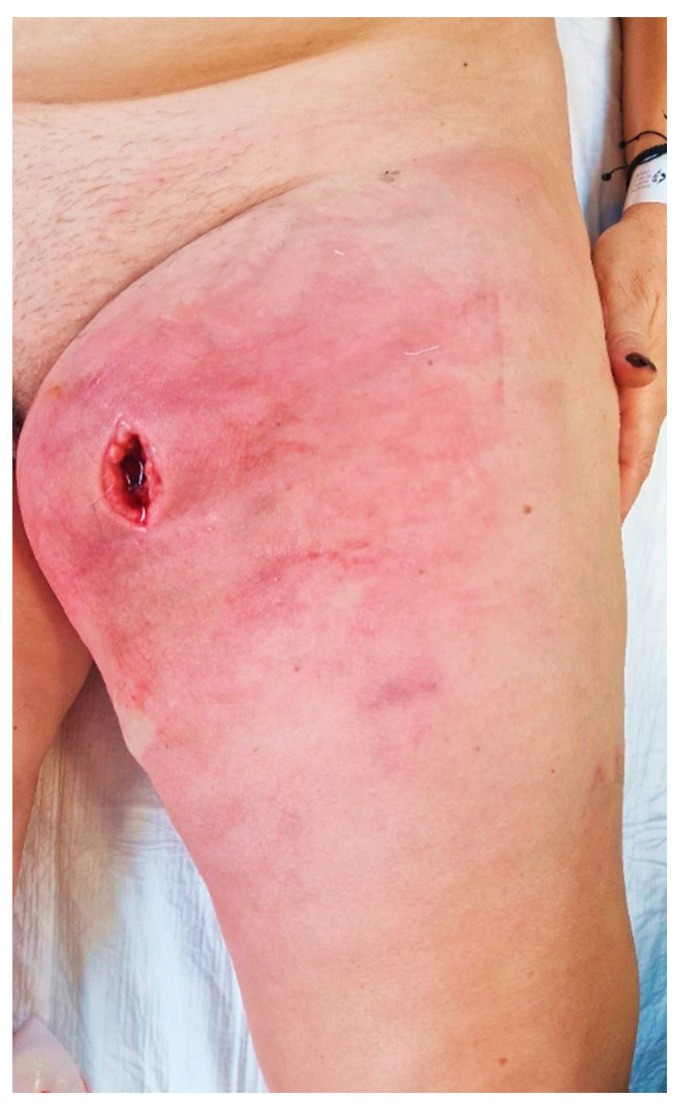
Aspect of thigh at presentation in the emergency department.

Upon admission to our emergency department, the patient had a high fever (39.3 °C), was shivering, and had tachycardia (120 bpm). At the clinical examination, she presented redness, swelling, and heat of the left thigh with an onset of 3 days prior, and a skin incision of approximately 3 cm, revealing a deep mass (Figure 1). A palpation showed in the anteromedial upper thigh an imprecisely delineated tumor, approximately 10/7 cm in size, adherent to the deep and superficial planes, associated with left inguinal lymphadenopathy, with palpable superficial nodes of 1–2 cm in diameter. Her past medical history was non-significant. The patient said she observed a slow-growing mass, progressively increasing for the past six months, after repeated minor trauma.

The blood tests at presentation showed high leukocytosis (46,000/µL) with neutrophilia, anemia (9.4 g/dL), high C-reactive protein level (120.64 mg/L), and hyperfibrinogenemia (470 mg/dL).

**Figure 2:**
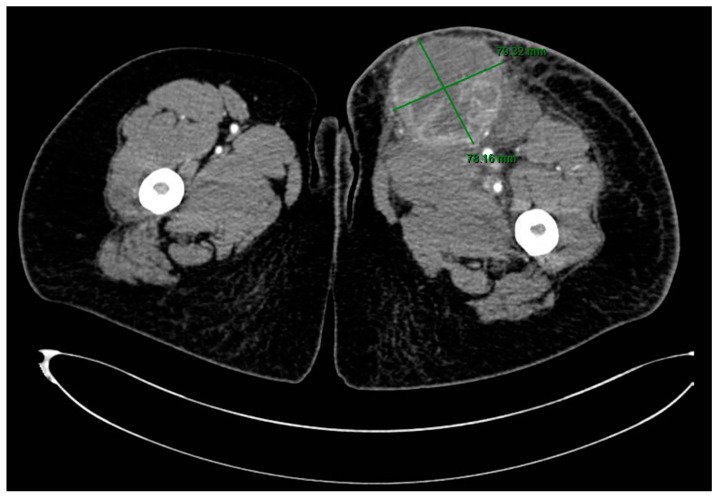
CT scan of the lower left limb showing a large mass in the upper thigh.

A CT scan of the thigh in an emergency setting showed a well-delineated, large mass of 8.6/7.7 cm, with extensive areas of necrosis, exhibiting intense and heterogeneous contrast enhancement, situated in contact posteriorly with the muscular planes, with densification of the perilesional subcutaneous cellular tissue and capturing inguinal lymphadenopathy up to 21.4 mm in diameter (Figure 2).

**Figure 3:**
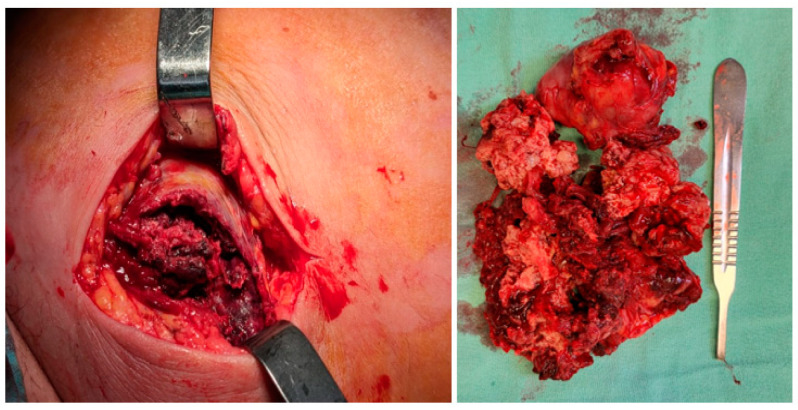
Intraoperative aspect of the mass.

An excisional biopsy of the mass was performed in an emergency setting, and the tumor was taken for histopathology evaluation. Intraoperatively, the mass appeared nonspecific, lacking a well-defined capsule, and was very friable, with no lateral or posterior cleavage. (Figure 3). The microbiological culture results from the wound biopsy and wound swabs were negative.

The frozen sections performed intraoperatively raised suspicion for malignancy; therefore, we suspected that the tumor formation was more likely a lymph node metastasis, and the patient underwent a CT scan with intravenous contrast to the brain, chest, abdomen, and pelvis, which showed no evidence of a primary tumor. A thorough dermatological examination did not reveal any primary lesions. While investigating the patient, no previous skin lesions or surgery history was elicited. A review of other systems, including genital examination and digital rectal examination, was negative. During the hospitalization, the patient refused to undergo an upper gastrointestinal endoscopy and a colonoscopy.

**Figure 4:**
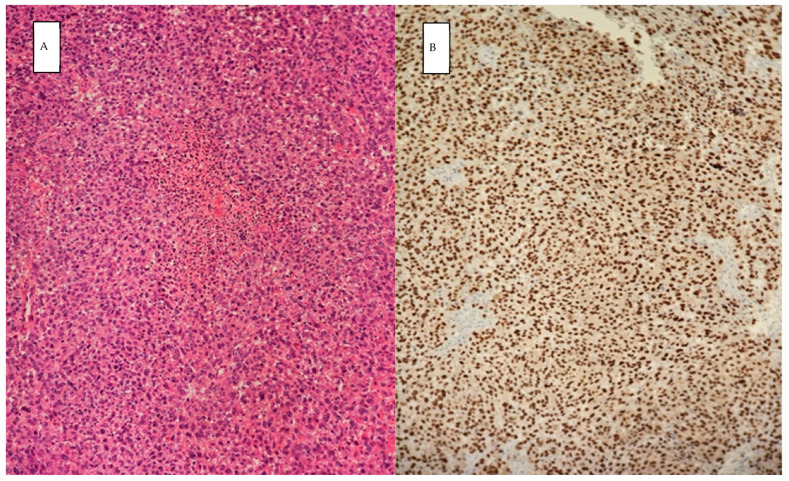
Histopathology: showing samples stained with hematoxylin and eosin under magnification of 20× (**A**), positive immunostaining for SOX10 (**B**) consistent with malignant melanoma.

The permanent histopathology report highlighted malignant tumor proliferation with large epithelioid and spindle cells, abundant eosinophilic cytoplasm, atypical nuclei, high mitotic rate (50 mitoses/10 HPF), and tumor necrosis (Figure 4). The tumor proliferation also involved lymphoid tissue. Immunohistochemistry tests showed the tumor was SOX 10 positive, melan-A positive, AE1/AE3 negative, WT1 negative, ER-negative, CD 31 negative CD34 negative, MSA negative, and CK19 negative; hence, a diagnosis of melanoma was established.

Broad-spectrum antibiotic treatment was continued, showing the remission of inflammatory signs in 5 days, allowing for the direct suture of the surgical wound.

She was discharged on the 14th day after surgery and was instructed to return for oncological evaluation in a tumor board. However, the patient did not show up for her appointment.

Clinicians must be aware of the varied clinical manifestations of malignant melanoma, especially in cases of occult melanoma where the primary site is not evident. The particularity of this case is the cellulitis/superinfected hematoma presentation of the patient, leading to prior surgery in the general surgery ward of another hospital. Previous surgery and local inflammation distorted the anatomy and the tumoral aspect. The patient was non-adherent despite being a registered nurse and did not attend the scheduled evaluation.

## 3. Discussion and Literature Review

### 3.1. Non-Cutaneous Malignant Melanomas

Non-cutaneous malignant melanomas are relatively uncommon compared to cutaneous malignant melanomas, with the two largest broad categories being ocular melanomas and mucosal melanomas, with additional reports of occurrence in less common sites, such as primary meningeal and adrenal melanomas [6].

Mucosal melanoma, accounting for about 1% of all diagnosed melanomas, develops from melanocytes in the mucosal membranes lining the respiratory, gastrointestinal, and urogenital tracts. Since these melanomas frequently occur in hidden locations, the subtle and late symptoms lead to delays in diagnosis and poor outcomes. Although most mucosal melanomas originate in the nasal cavity, accessory sinuses, oral cavity, anal canal, rectum, vulva, and vagina, they can virtually arise in nearly any part of the mucosal membranes, and data in the literature are continually growing on this topic [7,8]. Reported settings for gastrointestinal tract localizations are shown in Table 1, with more than 85% of primary gastrointestinal mucosa melanomas occurring in the anorectal and oropharyngeal regions [9]. The diagnosis is made by exclusion after ruling out metastasis from a cutaneous or unknown primary site [10]. Primary gallbladder malignant melanoma is an even rarer clinical entity, and its existence remains a controversial topic in the medical literature, with some questioning whether it is a distinct entity at all [11]. Primary pulmonary melanoma, or primary melanoma of the lung, is a very rare manifestation of melanoma arising from the bronchial epithelium, with around 40 cases reported in the English literature from 1916 to 2017 [12].

Uveal melanomas, rising from melanocytes in the uveal tract, are classified based on their location: anterior uveal melanomas originate in the iris, while posterior uveal melanomas develop in the choroid or ciliary body. The most common type is choroidal melanoma, accounting for nearly 90% of cases, followed by ciliary body melanoma (6%) and iris melanoma (4%) [13].

Primary malignant melanoma of the breast may derive from the breast skin or—less commonly—from the glandular parenchyma of the breast. Primary non-cutaneous malignant melanoma of the breast is exceedingly rare. Some authors suggest that the tumor may be metastatic from an unknown primary site or a primary tumor that has regressed, while others propose it could be a true primary tumor arising from ectopic melanocytes in the breast epithelium or through metaplastic transformation of a normal mammary duct precursor [14,15].

Primary adrenal melanoma is exceptionally rare and can arise due to the presence of ectopic melanocytes in the adrenal glands, which share a common embryological origin with adrenal medullary blasts at the neuroectodermal level, similar to the nervous system and skin [16].

Malignant primary melanocytic tumors of the central nervous system are believed to originate from melanocytes located in the leptomeninges and derived from neural crest cells. They can manifest as localized lesions, known as malignant melanoma, or as diffuse melanocytic lesions without forming macroscopic masses, referred to as melanomatosis. Malignant melanomas can arise at any point along the neuraxis, with a specific tendency to affect the spinal cord and posterior fossa [17].

**Table 1:** Non-cutaneous malignant melanoma sites as reported in the literature.

Gastrointestinal tract	Gastroesophageal junction primary melanoma	Hussein Agha Y et al., 2020 [18]
	Primary gastric melanoma	Sohail et al., 2024 [10], Schizas D et al., 2021 [9]
	Primary small bowel melanoma	Wu F et al., 2022 [19]
	Primary rectal melanoma	Ugonabo O et al., 2022 [20]
Urogenital tract	Primary gallbladder melanoma	Peison B et al., 1976 [21], Hatanaka N. et al., 1993 [22], Dong XD, et al., 1999 [23]
	Primary urethral melanoma	Nguyen Q et al., 2023 [24]
	Primary malignant melanoma of the uterine cervix	Duggal R et al., 2010 [25]
Respiratory tract	Primary pulmonary melanoma	Feng Y et al., 2016 [26], Filippini A. et al., 2015 [27], Dos Santos C. L et al., 2013 [28]
Uveal melanoma	Choroidal melanoma	Shields CL, 2014 [29], Singh P [30]
	Melanoma of the ciliary body	Shields CL, 2012 [31]
	Iris melanoma	Shields CL, 2012 [31]
Breast	Parenchymal melanoma without skin involvement	Drueppel D et al., 2015 [14], Koh J et al., 2019 [15]
Adrenal gland	Primary adrenal melanoma	González-Sáez L et al., 2011 [16]
Malignant primary melanocytic tumors of the central nervous system	Primary meningeal malignant melanoma	Lang-Orsini M et al., 2021 [17]
	Primary leptomeningeal melanomatosis (melanocytosis)	Noronha C et al., 2019 [32]

### 3.2. MUP Diagnosis

As already mentioned, MUP was first described by Das Gupta in 1963 and it can be diagnosed in patients diagnosed with melanoma upon histopathology, without any lesions on skin examination, ophthalmoscopy, or anus and genital examination, without a history of orbital enucleation or exenteration, and without the excision of any skin lesions [3]. Although the Das Gupta definition implies that a MUP can only be classified as such after several additional investigations and multidisciplinary consults, a study from Denmark recommends conducting only a thorough history and physical exam, as well as a CT/PET imaging study for staging purposes only, concluding that extensive screening for the primary tumor is redundant since they could only identify 1 potential primary tumor out of the 103 cases of MUP included in the study [33].

We performed a search throughout the literature for “melanoma unknown primary” on the Pubmed database, from January 2014 to May 2024, with a return of 792 articles. Out of these, we excluded those that were written in a non-English language, with 773 articles remaining. After a thorough screening of the titles and abstracts, we selected a final number of 118 articles that were relevant to the subject of our review. The reported sites for MUP and the clinical presentation are summarized in Table 2 and further detailed in the following sections.

Lymph nodes are the most frequently affected site in MUP, accounting for approximately 40–60% of MUP cases. In males, axillary and cervical involvement of lymph nodes is predominant, while females have an increased incidence of inguinal nodal involvement [34]. There are several studies in the literature documenting lymph node MUPs, usually with asymptomatic palpable lumps, but there have also been some cases with lower limb edema on presentation [35,36,37,38,39].

When it comes to extra-nodal sites in MUP, subcutaneous tissues are the most affected (30%), followed by the involvement of visceral organs (20%) [34].

Soft tissue involvement can present as hyper-pigmented nodules protruding through the skin with central ulceration and crusting. In these cases, there is no involvement of the epidermis, as the metastasis is located in the dermal layer, so any apparent skin lesions are the result of ulceration of the epidermis. The histopathology reports will therefore show melanocytes in the dermis, without continuation with the overlying epidermal layer. On the other hand, if the tumor does not cause any erosions of the overlying epidermal layer, it may present as a firm skin-colored swelling or a hypertrophic lesion [40,41]. Clinical differential diagnosis can be performed with posttraumatic conditions, benign soft tissue tumors, or other malignancies of the soft tissue, either common (lipoma, fibrolipoma) or more rarely encountered histopathological entities (sarcoma, solitary fibrous tumor etc.) [42].

The gastrointestinal tract is most commonly affected by MUP in the small bowel (51–71%), followed by the stomach (27%), colon (22%), and esophagus (5%) [43].

Averbukh et al. described a case of gastroesophageal junction involvement after an achalasia misdiagnosis on esophagogastroduodenoscopy (for which the patient received botulinum toxin injections in the cardiac sphincter). However, after a biopsy and further investigations, the diagnosis was MUP. The initial symptoms of presentation are mentioned in Table 2 [44]. Gastric MUPs may present clinically in various ways (Table 2), but most of the time they involve polypoid lesions and even diffuse polyposis [45]. There have even been reports of long-term survival in the literature, such as one case reported by Takahashi et al. on a 10-year survivor after the diagnosis of gastric MUP [46]. The high rates of intestinal MUPs among its gastrointestinal presentations are most probably due to its hematogenous metastasis with invasion of the bowel [47]. In particular, the small intestine is the most common site primarily due to its abundant blood supply. Intestinal MUP can present with a gastrointestinal hemorrhage and small bowel obstruction or intussusception in acute settings [48,49]. However, intestinal MUPs may also present in a non-acute setting, with symptoms such as vomiting, weight loss, and abdominal pain [40]. Despite not being as common as small bowel involvement, the large bowel might also be a site for MUPs. For example, Reddy et al. described a case of colon involvement after a previous parotidectomy for a parotid gland MUP [50].

Symptoms such as nausea, weight loss, abdominal distension, etc. (see Table 2) can also be present when MUP affects the liver, which is mostly diagnosed in a non-acute setting [51,52]. Sometimes, hepatic involvement can even be diagnosed as an incidental finding during routine check-ups [53]. However, Tiong et al. described a case of ruptured hepatic MUP, where a hemorrhage from the lesion generated an acute presentation of right upper quadrant pain [54]. Although very rare, even the gallbladder can be affected by MUP, with 19 cases in almost 30 years reported in a review from the Duke University Medical Center from 1999 [22], with some patients presenting with acute cholecystitis and hemobilia [55]. Upper abdominal pain, fever, and jaundice associated with cholestatic syndrome might indicate pancreatic MUP [56].

Brain tumors can also prove to be MUPs, either individually or along with other sites. As a 10-year study from the Netherlands showed, 84.7% of brain MUPs were found in patients who also presented other metastases, while 15,3% were solely brain metastases and even though the overall survival increased over time, it rarely exceeded 12 months [57]. Presentations are very different, depending on the involved cortical area, with reports describing patients presenting with dizziness, headaches, diplopia, seizures, ataxia, or symptoms of hydrocephalus (severe nausea and vomiting) [58,59]. However, it was observed that the frontal lobe is most frequently involved in melanoma brain metastases because of the high vascularity in this region, often being responsible for psychiatric symptoms [60]. MUP diagnosed in the brain does not necessarily have to present as a solitary brain tumor since the leptomeningeal disease was described as a possible clinical scenario of MUP brain involvement [61]. The nervous system can be affected by MUP not only in the central nervous system because spinal nerve root involvement has also been cited in the literature. Symptoms range from simple limb tingling and numbness [62] to cauda equina syndrome when multiple intradural extramedullary lesions are present in the spine, sometimes associated with central nervous system involvement, such as the lateral ventricles or posterior fossa [63].

Although very rarely reported outside the lungs, intrathoracic sites of MUP are also possible. Pujani et al. identified a mediastinal MUP through a Tru-cut biopsy in a patient who showed a lack of symptom improvement after oncological treatment for an adenocarcinoma misdiagnosed through CT-guided fine needle aspiration cytology from the same mass 6 months prior. Initially, the patient presented with a dry cough, chest pain, and weight loss [64]. We also found a single case of cardiac MUP presenting as a large mass of the right atrium, causing a subsequent ejection fraction of 40%. After the removal of the cardiac MUP, the atrial reconstruction was performed by using a pericardial patch [65]. However rare, the most common intrathoracic site reported in the literature is the lung. Usually, pulmonary MUP presents as a solitary nodule in imaging studies and it may even invade the neighboring structures, such as the costal arches [66], or be accompanied by satellite neighboring structures nodules, such as intramuscular lesions [67]. An interesting presentation is Horner’s syndrome, when the MUP affects the apical segment of the upper pulmonary lobe, hence compressing the stellate ganglion of the sympathetic nervous system. Gebauer et al. described a patient who presented with eyelid ptosis and thoracic pain, without miosis or anhidrosis, thus only partially displaying the symptoms of Horner’s syndrome [68]. Aside from biopsy, MUP can be diagnosed through the histopathologic analysis of pleural fluid cytology [69].

Another scarcely cited MUP site throughout the literature is the respiratory tract. While nasopharyngeal MUP might generate chronic cerumen impaction, recurrent severe epistaxis, as well as diminished hearing [70], endobronchial MUP presents with cough and hemoptysis [71].

There are only a few articles in the literature reporting on patients with melanuria as the first presentation of MUP [72]. Hematospermia has also been reported as a first symptom in a case of seminal vesicle MUP, a rare occurrence with only a few cases cited in the literature [73,74].

The incidence of melanoma in pregnancy has been estimated to range from 0.14 to 2.8 per 1000 live births and it accounts for about 8% of all tumors arising during pregnancy. The placenta is a very uncommon site for metastases, although this occurrence has been noted in the literature. Garcia-Ramiu et al. described a case of synchronous brain and placenta MUP in a pregnant patient who underwent brain surgery a few days after delivery. The histopathology report on the placenta identified melanocytes on the mother side of the placenta, in the intervillous space. Thus, because the intravillous space was not invaded by melanocytes, the newborn was presumed to not be affected and was closely monitored throughout their first years [59]. A study on transplacental metastases in infants reported that most of the cases were due to melanoma: 27 patients out of 87 cases of placental or fetal metastases were attributed to melanoma. Furthermore, 22% of the cases of melanoma placental metastasis affected the fetus, with five out of the six fetuses dying from this disease. The mechanisms underlying the transplacental spread of melanoma are not understood, but it is presumed that the production of growth and angiogenic factors in this organ, as well as its complex blood supply and lower immune response on the fetal side, may play a role in the process [59,75].

MUP can be identified in the bones, as well. Although any bones may be invaded, such as the skull, rib cage, sternum, clavicles, humeri, scapulae, or femora, as shown by Tang et al. [76], the spinal vertebrae are the most common site. The symptoms are not very different from those affecting the peripheral nervous system at the level of the spinal nerve roots, because the physiopathology is the same (i.e., spinal root compression), the only difference being that vertebral MUP is a metastasis that develops inside the bone tissue, thus presenting lower limb radiculopathy, lower back pain, dysuria, motor deficit, and paresis [77,78,79]. When the bone marrow is involved, general or hematological symptoms are more prominent, such as general weakness, loss of appetite, vomiting, and paraclinical indicators such as progressive thrombocytopenia should also catch the clinician’s attention [80,81].

The muscular system may sometimes be invaded by MUP, although solitary muscle involvement is relatively unusual because of the muscle hostility for cancer development [82]. Usually, muscular MUP presents as a lump in the respective region, such as in the buttock if the gluteus maximus muscle is involved or in the temporal region when the temporal muscle is invaded by MUP [82,83]. Other times, on top of the mass on palpation, symptoms such as trismus may be present in the case of temporal muscle MUP [84]. When the limbs are involved, antalgic positions can be noted. For example, in cases of triceps brachii MUP, semi-flexion of the forearm and hand flexion has been observed [85].

Metastatic melanomatous lesions of the breast may be asymptomatic or present as palpable, dense, and well-circumscribed nodules in physical examination. Metastasis to the breast commonly appears in the upper outer quadrant as single or multiple circumscribed round masses with slightly irregular margins and various sonographic morphologies. Metastatic lesions typically do not present tumor spiculation, microcalcification, or secondary skin or nipple changes [86].

MUPs of the salivary glands can present as an asymmetric swelling of the submandibular region associated with snoring complaints when the submandibular gland is concerned [87]. In comparison to parotid melanoma with known primary, parotid parenchyma is much more likely to be the site of parotid MUP. Although parotid MUP is more frequently associated with stage IV disease and develops metastatic disease more quickly during follow-up, the overall survival between patients with parotid MUP and those with parotid melanoma of known origin was similar when presenting with stage-matched disease [88]. Adrenal glands can be affected by MUP as well, with patients presenting with symptoms such as asthenia and weight loss, recent-onset vitiligo, and dorsal pain. When faced with a histopathological dilemma, the presence of melanoma in both adrenal glands advocates for metastatic melanoma rather than primary adrenal melanoma [89,90].

Choroidal MUP is a rather rare presentation of non-uveal melanoma that can mimic primary uveal melanoma, with symptoms such as the progressive loss of vision. Although examination can show a choroidal lesion with clinical and sonographic characteristics concerning primary uveal melanoma, histopathology and further analyses are the ultimate factors for definitive diagnosis. If genetic analysis manages to identify the Val600Glu (c.1799T>A) *BRAF* mutation, the finding is consistent with choroidal MUP, because it indicates a primary lesion on the skin [91].

Sometimes, MUPs of various locations can generate paraneoplastic presentations. Mondragon et al. reported a case of opsoclonus–myoclonus syndrome, with only five cases previously cited in the literature. They reported on a patient who also suffered disabling holocranial headache, sudden loss of consciousness, aggressive behavior, and vertigo and was ultimately diagnosed postmortem with lymph node MUP [92]. Another case report, by Rahimi et al., described a case of paraneoplastic vitelliform maculopathy presenting with progressive bilateral vision loss and photophobia, eventually being linked to multiple-site MUPs (intra-abdominal lymph nodes, small bowel, lung, and central nervous system) [93].

**Table 2:** MUP sites and clinical presentation as reported in the literature.

MUP Site		Clinical Presentations	References
Lymph nodes		Asymptomatic palpable lump Limb edema Superior vena cava syndrome (facial edema, collateral venous circulation in the neck)	Nakamura et al., 2023 [35] Doyle et al., 2023 [36] Phan et al., 2021 [37] Bankar et al., 2015 [38] Eltawansy et al., 2015 [39] Andrianandrasana et al., 2023 [82]
Soft tissues		Ulcerated skin nodules Subcutaneous swellings Hypertrophic lesions Numbness and pain in the limbs	Babu et al., 2022 [40] Sirvan et al., 2019 [41] Spoto et al., 2018 [94] Liu et al., 2024 [95]
Gastro-intestinal tract	Gastroesophageal junction	Progressive dysphagia	Averbukh et al., 2019 [44]
	Stomach	Vomiting Early satiety Anorexia Unintentional weight loss Anemia Nausea Fatigue	Yan et al., 2023 [45] Takahashi et al., 2020 [46] Myrou et al., 2021 [96] Cortellini et al., 2021 [97]
	Intestines	Gastrointestinal hemorrhage Bowel obstruction, intussusception Vomiting Weight loss Abdominal pain	Sirvan et al., 2019 [41] Vrable et al., 2017 [48] Wu et al., 2022 [49] Reddy et al., 2014 [50] Mui et al., 2019 [98] De Monti et al., 2018 [99] Stagnitti et al., 2014 [100]
	Pancreas	Upper abdominal pain Fever Jaundice	Ben Slama et al., 2017 [56]
	Liver	Asymptomatic Hepatomegaly Ascites Lower limb edema Abdominal distention Loss of appetite Nausea Weakness Progressive weight loss Fever Sweating Upper right quadrant pain	Yan et al., 2023 [45] Yuan et al., 2023 [51] Wang et al., 2023 [52] Cheng et al., 2021 [53] Tiong et al., 2023 [54]
	Gallbladder	Acute cholecystitis Hemobilia	Dong et al., 1999 [23] Onozawa et al., 2014 [55]
Nervous system	Central nervous system	Dizziness Headaches Diplopia Seizures Visual auras Ataxia Acute confusion Magnetic gait Muscle weakness Urinary incontinence Hydrocephalus (nausea, vomiting) Leptomeningeal disease Weight loss	Doyle et al., 2023 [36] Padilla et al., 2023 [57] Nguyen et al., 2022 [58] Garcia-Ramiu et al., 2022 [59] Sawalha et al., 2023 [61] Kuriakose et al., 2015 [65] Mremi et al., 2021 [101] Takagi et al., 2020 [102]
	Peripheral nervous system	Paresthesia Numbness Paraplegia Cauda equina syndrome	Naing et al., 2004 [62] Chen et al., 2022 [63] Takagi et al., 2020 [102]
Mediastinum		Dry cough Chest pain Weight loss	Pujani et al., 2017 [64]
Heart		Dyspnea Fatigue Weight loss	Kuriakose et al., 2015 [65]
Lung		Cough Thoracic pain Horner’s syndrome	El Haj et al., 2021 [66] Tsaknis et al., 2021 [67] Gebauer et al., 2020 [68] Yamamoto et al., 2023 [69]
Respiratory tract	Nasopharyngeal	Cerumen impaction Epistaxis Impaired hearing	Azoury et al., 2015 [70]
	Endobronchial	Cough Hemoptysis	Kim et al., 2023 [71]
Genitourinary system		Melanuria Hematospermia	Diamantopoulos et al., 2023 [72] Meng et al., 2000 [73] Fabiani et al., 2016 [74]
Placenta		-	Garcia-Ramiu et al., 2022 [59]
Bones		Radiculopathy Lower back pain Dysuria Motor deficit Paresis	Tang et al., 2019 [76] Kakutani et al., 2008 [77] Mathew et al., 2017 [78] Tang et al., 2020 [79]
Bone marrow		General weakness Loss of appetite Vomiting Progressive thrombocytopenia	Matsumoto et al., 2021 [80] Suzuki et al., 2014 [81]
Muscles		Lumps Trismus Antalgic positions of limbs	Tsaknis et al., 2021 [67] Andrianandrasana et al., 2023 [82] Grech et al., 2020 [83] Dalle Carbonare et al., 2017 [84] Rastrelli et al., 2014 [85]
Breast		Asymptomatic Palpable nodule	Kim et al., 2017 [86] Agosto-Arroyo et al., 2017 [103] El-Tani et al., 2016 [104]
Salivary glands		Asymmetric swelling Snoring complaints	Gorris et al., 2021 [87] Scott et al., 2016 [88]
Adrenal glands		Asthenia Weight loss Recent-onset vitiligo Dorsal pain	Drouet et al., 2017 [89] Blanco et al., 2014 [90]
Choroid		Progressive loss of vision	Rieth et al., 2021 [91]
Paraneoplastic presentations		Opsoclonus–myoclonus syndrome Paraneoplastic vitelliform maculopathy	Mondragón et al., 2019 [92] Rahimi et al., 2018 [93]

### 3.3. Staging and Prognosis

The question of whether lymph node melanoma should be classified as MUP or primary melanoma was raised by Tchernev et al., especially in cases when only one node is involved. They suggest the hypothesis that ectopic melanocytes, which are known to be present in lymph nodes or other visceral organs, could undergo malignant transformation similar to the cutaneous melanocytes, as a result of oncogenic stimuli exposure or genetic predisposition. Thus, the staging of single lymph node MUP should be performed similarly to the first two stages of cutaneous melanoma, but strictly referred to the primary melanoma of the lymph nodes. On the other hand, should several lymph nodes be affected, this finding should be translated as a definite marker for metastatic melanoma. Tchernev et al. suggest that the involvement of a single lymph node is rather more suggestive of a primary lymph node melanoma, which does not require a very aggressive therapeutic approach in cases with small tumor thickness [105].

Regardless of these debates, the American Joint Committee on Cancer (AJCC) developed guidelines for MUP staging. Per the eighth edition of the AJCC staging criteria, patients with melanoma metastases in the subcutis, soft tissues, and/or lymph nodes and without a detectable primary tumor are directly diagnosed with stage III disease. On the other hand, patients with distant metastases, including visceral ones, are directly diagnosed with stage IV disease [106].

When it comes to prognosis, there are some contradicting studies on whether patients with MUP have better overall survival times than patients with primary melanoma. A retrospective study from Belgium, conducted over 14 years, compared the survival times of patients diagnosed with metastatic primary cutaneous melanoma and patients diagnosed with MUP. The patients with metastatic primary cutaneous melanoma had a median survival time (years) of 1.5 (95% CI: 1.1–1.8) in 2004–2008, 1.1 (95% CI: 0.8–1.5) in 2009–2013, and 1.6 (95% CI: 1.3–2.4) in 2014–2017, respectively. On the other hand, patients diagnosed with MUP showed improving survival (years) over time, with a median survival time of 2.0 (95% CI: 1.4–2.9) in the most recent period, 1.1 (95% CI: 0.7–1.3) in 2009–2013, and 0.9 (95% CI: 0.6–1.2) in 2004–2008 [107]. A similar result was reported by another study on 2706 patients which included 2321 (85.8%) patients with primary cutaneous melanoma and 385 (14.2%) with MUP. MUP patients were more likely to have advanced and metastatic disease in their initial presentation. In an adjusted analysis, patients with MUP had a superior overall survival, in comparison to patients with advanced and metastatic primary cutaneous melanoma, although they had poorer prognostic characteristics upon presentation [108]. On the other hand, Rassy et al. showed different results in their study. They included 377 patients with primary cutaneous melanoma and 85 patients with MUP. The overall survival time of primary melanoma patients was 49 months, while MUP patients had an overall survival time of 44 months, which was not statistically significant [109].

Several reviews of the literature show that better survival for MUP patients was observed for MUP patients in stages III and IV, as opposed to those with corresponding stages of primary melanoma, but not all studies showed statistical significance for these findings. Various prognostic factors identified throughout the literature are not unanimously agreed upon. For example, several studies show that the lower the number of involved lymph nodes, the better the outcome, while other studies could not confirm this hypothesis. The same is true for allegedly better survival depending on gender. There is no consensus on an age cutoff or the extent of lymphadenectomy regarding survival. While surgical treatment is universally accepted as a favorable measure to be taken to increase overall survival, the same cannot be said for oncological treatments, which were correlated with lower survival times, albeit not statistically significant [2,33,110].

### 3.4. Treatment

Surgery is a pivotal part of the treatment of MUP, since it improves patients’ overall survival. Surgical resection for curative purposes might involve lymphadenectomy, craniotomies followed by excision, lung resections, or bowel resections. In more advanced cases, surgery might be performed for palliative purposes and the remission of acute symptoms. Even in stage IV MUP, a better survival rate has been proven when surgery was performed to remove the metastases [2,111].

For patients with lymph node MUP who are good surgical candidates, the standard is lymph node dissection, with a recurrence rate of 11%, sometimes associated with parotidectomy if the parotid is involved [33,112]. It has been proposed that subcutaneous or soft tissue MUP be treated by wide local excision with margins at 1–2 cm, despite the higher overall recurrence rates (65–78%) [112].

However, surgical treatment should always be completed by oncological treatment, such as chemotherapy, radiotherapy, and/or immunotherapy, either as adjuvant treatment or for palliative purposes [33]. In the pre-novel therapy era, there was a wide range of possible regimens following surgery, such as radiotherapy, cytotoxic chemotherapy (cisplatin, dacarbazine, procarbazine, methotrexate, vincristine, lomustine, etoposide, thio thepa, methyl lomustine, estramustine, carmustine, etc.) or immunotherapy (interferon-α, interleukin-2) or a combination of these [2].

Nowadays, novel therapies are implemented, and targeted immunotherapy regimens are becoming more popular. Since 2011, the Food and Drug Administration (FDA) and the European Medicines Agency (EMA) have approved several systemic novel therapies. They are represented by immunotherapy (immune checkpoint inhibitors) or targeted therapy (BRAF and/or MEK inhibitors) [2]. Immune checkpoint inhibitors are monoclonal antibodies designed to boost anti-tumor T-cell responses by counteracting the suppression caused by immune checkpoints. These checkpoints include cytotoxic T-lymphocyte-associated protein 4 (CTLA-4), targeted by antibodies like ipilimumab, and the programmed death-1 (PD-1) receptor, targeted by antibodies such as nivolumab and pembrolizumab. Targeted therapy operates through a distinct mechanism by inhibiting cancer cell proliferation. It employs selective BRAF inhibitors, such as vemurafenib, dabrafenib, and encorafenib, as well as MEK inhibitors, including trametinib, cobimetinib, and binimetinib [108].

In 2011, ipilimumab, the first immune checkpoint inhibitor targeting CTLA-4, received approval. This was followed by the approval of BRAF inhibitors vemurafenib in 2012 and dabrafenib in 2013, and the MEK inhibitor trametinib in 2014. In 2015, PD-1 blocking antibodies nivolumab and pembrolizumab were approved, as well as the combined BRAF/MEK inhibitors dabrafenib plus trametinib and vemurafenib plus cobimetinib. The combination of ipilimumab and nivolumab was approved in 2016. It is important to note that immunotherapy is available to all patients, regardless of mutation status, while targeted therapy is restricted to patients with mutations in the BRAF gene. Although BRAF/MEK inhibitors are classified as targeted therapies, they also seem to provoke immune responses in melanoma by altering the tumor microenvironment and immune surveillance [2,113].

As shown in a systematic review by Boussios et al. in 2021, even in the post-novel therapy era, local therapy is the treatment of choice in 47.7% of cases. On the other hand, novel first-line therapy has taken hold and is now used in stage III and IV MUP in 27.8% of cases, including immunotherapy or targeted therapy alone, or a combination of both. Despite these advancements, cytotoxic chemotherapy is still being used, but only in 9% of patients with MUP [2].

For stage III MUP, a one-year adjuvant regimen of nivolumab or pembrolizumab is generally recommended. When the BRAF mutation is present or when active autoimmune diseases prohibit immunotherapy with checkpoint inhibitors, targeted agents such as dabrafenib or trametinib may be a good alternative. Stage IV MUP, on the other hand, requires a rather complex regimen of treatment, combining not only surgery and immunotherapy, as was the case for stage III MUP, but also chemotherapy and/or radiotherapy, making the treatment a lot more aggressive [2].

Unfortunately, there is no consensus yet on whether these novel therapies improve survival. A study published in 2017 concluded that MUP patients have worse outcomes under immunotherapy as compared to those with primary cutaneous melanoma. On the other hand, another study published in 2019 showed that MUP patients are great candidates for therapies with checkpoint inhibitors [114,115]. A phase 2 trial on the treatment of advanced melanoma, including some MUP cases, published in 2023 in the New England Journal of Medicine, proved that three doses of neoadjuvant pembrolizumab, followed by surgery and then 15 doses of adjuvant pembrolizumab, provide a longer event-free survival than 18 doses of adjuvant pembrolizumab only (event-free survival at 2 years was 72% vs. 49%, respectively) [116]. Studies like this one are necessary for MUP patients if only in order to establish the true impact of the novel therapies on the overall survival of these patients.

## 4. Conclusions

Melanoma of unknown primary (MUP) represents a unique and challenging subset of melanoma cases, characterized by metastatic disease without an identifiable primary lesion. Our case report and literature review highlight the diverse clinical manifestations and diagnostic challenges associated with MUP, emphasizing the necessity for a thorough clinical examination, comprehensive imaging, and histopathological evaluation. Due to its rarity and atypical presentations, MUP often poses significant diagnostic and therapeutic challenges. Understanding the genetic and molecular features, epidemiology, and prognostic factors of MUP is essential for optimizing patient care. Advances in molecular biology and imaging techniques may provide new insights into the origins and behavior of MUP, potentially leading to more effective diagnostic and therapeutic strategies. Clinicians must maintain a high index of suspicion for MUP in patients presenting with metastatic melanoma without a known primary site, and a multidisciplinary approach is crucial for accurate diagnosis and optimal management.

## Data Availability

Datasets are available on request from the authors.
